# Radiotherapy Response Prediction in Myxofibrosarcomas and Undifferentiated Soft Tissue Sarcomas Using DNA Methylation and Copy Number Profiling

**DOI:** 10.1177/10668969251412902

**Published:** 2026-01-29

**Authors:** Tony G. Kleijn, Baptiste Ameline, Wierd Kooistra, Léon C. van Kempen, Gilles F.H. Diercks, Robert J. van Ginkel, Lukas B. Been, Barbara L. van Leeuwen, Anna K.L. Reyners, Thomas C. Kwee, Paul C. Jutte, Joris J.W. Ploegmakers, Henk Bijl, Bart Vanhauten, J. Fred Ubbels, Ed Schuuring, Albert J.H. Suurmeijer, Jacco J. de Haan, Daniel Baumhoer, Arjen H.G. Cleven

**Affiliations:** 1 Department of Pathology and Medical Biology, University Medical Center Groningen, 10173 University of Groningen, Groningen, The Netherlands; 2 Department of Pathology, Amsterdam University Medical Center, Amsterdam, The Netherlands; 3 Bone Tumor Reference Center at the Institute for Medical Genetics and Pathology, 30262 University Hospital Basel, Basel, Switzerland; 4 Department of Pathology, 30262 University Hospital Antwerp, University of Antwerp, Antwerp, Belgium; 5 Department of Surgery, 10173 University Medical Center Groningen, University of Groningen, Groningen, The Netherlands; 6 Department of Medical Oncology, 10173 University Medical Center Groningen, University of Groningen, Groningen, The Netherlands; 7 Department of Radiology, 10173 University Medical Center Groningen, University of Groningen, Groningen, The Netherlands; 8 Department of Orthopedic Surgery, 10173 University Medical Center Groningen, University of Groningen, Groningen, The Netherlands; 9 Department of Radiotherapy, 10173 University Medical Center Groningen, University of Groningen, Groningen, The Netherlands; 10 Basel Research Centre for Child Health, Basel, Switzerland

**Keywords:** myxofibrosarcoma, undifferentiated soft tissue sarcoma, DNA methylation, copy number variation, profiling, radiotherapy response

## Abstract

**Background:**

Myxofibrosarcoma (MFS) and undifferentiated soft tissue sarcoma (USTS) are common sarcoma subtypes with overlapping molecular features. Both are treated with neoadjuvant radiotherapy followed by surgery, yet radiotherapy response is variable and unpredictable. This study investigated DNA methylation and copy number variation (CNV) profiles obtained from pre-radiotherapy biopsies as predictive biomarkers of radiotherapy response.

**Patients and methods:**

Pre-radiotherapy biopsies and post-radiotherapy resections were obtained from 49 patients (27 MFS, 22 USTS). Radiotherapy response was assessed on the resection specimens using the EORTC-STBSG 5-tier system; grades A-C (<10% viable tumor) were classified as responders, D-E (≥10% viable tumor) as non-responders. Genome-wide DNA methylation and CNV data were generated from the pre-radiotherapy biopsies using Illumina MethylationEPIC BeadChips and were correlated with response grades.

**Results:**

DNA methylation profiling yielded evaluable results in 23/49 tumors (15 MFS, 8 USTS), with 9 responders and 14 non-responders. Unsupervised methylation clustering, incorporating public datasets, showed that MFS, USTS, and pleomorphic liposarcomas formed a single, heterogeneous cluster. Similarly, CNV profiles did not distinguish MFS from USTS. Methylation patterns did not significantly differ between responders and non-responders. CNV profiles were largely comparable between responders and non-responders, except of a significantly higher frequency of chromosome 11q24.1 loss in responders compared to non-responders (100% vs 33%; P = 0.0039).

**Conclusions:**

Our findings support the concept that MFS and USTS represent a spectrum of the same disease. We could not demonstrate the value of DNA methylation profiling in radiotherapy response prediction. However, 11q24.1 loss may represent a potential predictive biomarker and merits further validation.

## Background

Myxofibrosarcoma (MFS) and undifferentiated soft tissue sarcoma (USTS) are common sarcoma subtypes, primarily arising in the extremities of elderly patients.^1^ MFS was historically regarded as a subtype of USTS (“myxoid malignant fibrous histiocytoma”) but is now recognized as a distinct entity based on its unique clinicopathologic and radiologic characteristics.^2,3^ Histologically, MFS is composed of spindle to polygonal cells exhibiting nuclear atypia, embedded within a myxoid stroma and associated with curvilinear, slit-like vasculature. It exhibits a high rate of local recurrence and tendency to recur with increased histologic grade. In contrast, USTS is generally more cellular and of higher grade and has a greater tendency to develop distant metastases. MFS has generally a better prognosis than USTS, with Fédération Nationale des Centres de Lutte Contre le Cancer (FNCLCC) grading being a predictor of metastasis and tumor-related death.^2^ Despite these differences, MFS and USTS are molecularly largely indistinguishable, except for higher matrix-associated gene expression in MFS, suggesting they rather represent a spectrum of the same disease than separate entities.^4^ Both are characterized by complex chromosomal aberrations, underscoring the pivotal role of chromosomal instability in the pathogenesis and progression of these aggressive malignancies.^5,6^

The standard treatment for patients with non-metastatic MFS and USTS is neoadjuvant radiotherapy followed by surgical resection. Neoadjuvant radiotherapy has been shown to significantly improve disease-free survival and provides potential operative advantages.^7,8^ Nevertheless, the therapeutic benefits of radiotherapy must be weighed against the risk of adverse effects and wound-healing complications.^9^ Patient responses to radiotherapy vary considerably. Therefore, identifying predictive biomarkers of response to radiation to personalize the use of radiotherapy would be beneficial in reducing toxicities and improving quality of life in long-term cancer survivors.^10^ To date, no reliable method exists to predict the response to radiotherapy in MFS or USTS.

Emerging data from various cancer types indicate that genomic profiling, including DNA methylation profiling and copy number variation (CNV) analysis, not only serve as diagnostic tools but also hold promise as predictors of treatment response, including to radiotherapy.^10–13^ Altered gene expression in tumor cells due to DNA methylation or CNVs can influence radiotherapy sensitivity. For example, gain-of-function alterations in genes involved in DNA damage repair due to hypomethylation or gene amplification are hypothesized to contribute to reduced radiosensitivity.^13^ As an example, increased *EGFR* expression contributes to radio resistance in different solid human tumors by activating the RAS-RAF-MEK-ERK pathway, which promotes homologous recombination repair and evasion of apoptosis.^14^ In MFS and USTS, the utility of DNA methylation profiling and CNV analysis as predictive biomarkers for response to neoadjuvant radiotherapy is so far unknown.

The aim of this study was to explore DNA methylation and CNV patterns in MFS and USTS obtained from pre-radiotherapy biopsy specimens to identify potential predictive biomarkers for response to neoadjuvant radiotherapy.

## Patients and Methods

### Patient Selection

This study was approved by the Medical Ethics Committee of the University Medical Center Groningen (UMCG), an expert sarcoma center in the Netherlands. Archival pre-radiotherapy biopsies and post-radiotherapy resection specimens were retrieved from the OncoLifeS (Oncological Life Study) biobank and the UMCG pathology archive. Tumors were graded using the FNCLCC system and pseudonymized in accordance with the Code of Conduct for Health Research (www.coreon.org). Two expert sarcoma pathologists (A.C. and A.S.) reviewed histology, immunohistochemistry, and molecular data to confirm diagnoses. In line with the WHO criteria, MFS and USTS were defined by their histological features together with an absence of immunohistochemical or molecular features indicative of a distinct entity or cell lineage.^1^ None of the diagnoses were revised. Patient clinical data, including age, sex, primary tumor site and size, risk grade, treatment, pathologic response, and clinical outcome, were extracted from the electronic patient record system. Patients who did not complete radiotherapy were excluded.

### Pathologic Response Assessment

Radiotherapy response was assessed on the resection specimens according to the European Organization for Research and Treatment of Cancer-Soft Tissue and Bone Sarcoma Group (EORTC-STBSG) 5-tier response score system: A (no viable tumor cells), B (single viable tumor cells or small clusters, < 1% of the specimen), C (≥1% to <10% viable tumor cells), D (≥10% to <50% viable tumor cells), and E (≥50% viable tumor cells).^15^ One representative central tumor slab from each resection was entirely embedded in a grid-like manner for evaluation. Pathologic response was defined as <10% viable tumor cells (response grades A-C), and non-response as ≥10% (response grades D-E).^15^

### DNA Methylation Profiling

Genomic DNA was extracted from formalin-fixed paraffin-embedded (FFPE) pre-radiotherapy biopsy specimens, using representative tumor tissue with a tumor cell content of at least 70%. DNA extraction was performed using the QIAamp DNA FFPE Tissue Kit (QIAGEN) according to the manufacturer's instructions. DNA concentrations were quantified using a Qubit Fluorometer. Only biopsy specimens yielding ≥100 ng of genomic DNA were included for methylation analysis.

Genome-wide methylation data were generated using the Illumina Infinium Human MethylationEPIC v1.0 BeadChip and its successor v2.0 BeadChip, as previously described.^16^ The Illumina FFPE DNA Restoration Kit was applied in a subset of tumor samples (n = 11) according to the manufacturer's protocol.

Raw intensity data (IDAT) files from the MethylationEpic BeadChips were processed using the R-package “minfi” (https://bioconductor.org/packages/minfi/). The “convertArray” function from “minfi” was manually adapted to convert EPIC v2.0 arrays into a virtual EPIC v1.0 array to enable joint normalization and data processing from both platforms. Probes associated with known single-nucleotide polymorphisms, non-CpG sites, or sex chromosomes were excluded. Samples with a mean detection *P* > 0.03 were discarded. Batch effect correction was applied to the beta values using the R-package “ChAMP” (https://bioconductor.org/packages/ChAMP/) to eliminate bias related to array type (EPIC v1.0 and v2.0) and whether the DNA was restored using the Illumina FFPE DNA Restoration Kit.

Unsupervised clustering was performed using Uniform Manifold Approximation and Projection (UMAP), with and without integration of publicly available datasets, to respectively support diagnostic classification and to identify potential methylation-based predictors of radiotherapy response. UMAP was performed on the results of a principal component analysis (PCA) calculated on the top 10 000 most variable CpG sites, using the singular value decomposition of the beta matrix. UMAP graphs were generated with the R package “uwot” (https://github.com/jlmelville/uwot). The settings used to generate the non-linear regression model were PCA = 40, neighbors = 8; other parameters remained default.

For comparison, 3 MFS and 2 pleomorphic liposarcomas (PLS) from the UMCG, and publicly available datasets (GSE140686, and E-MTAB-9875)^11,17^ of 40 angiosarcomas (AS), 16 atypical fibroxanthomas and pleomorphic dermal sarcomas (AFX/PDS), 46 melanomas of the skin (MEL), 45 dermatofibrosarcoma protuberans (DFSP), 29 epithelioid sarcomas (ES), 34 leiomyosarcomas (LMS), 17 low-grade fibromyxoid sarcomas (LGFMS), 30 malignant peripheral nerve sheath tumors (MPNST), 27 MFS, 38 myxoid liposarcomas (MLPS), 25 PLS, 96 alveolar rhabdomyosarcomas (RMS (ALV)), 72 rhabdomyosarcomas embryonal (RMS (EMB)), 24 rhabdomyosarcomas *MYOD1*-mutated (RMS (MYOD1)), 22 squamous cell carcinomas of the skin (SCC), 60 synovial sarcomas (SYSA), 33 well-differentiated/dedifferentiated liposarcomas (WDLS/DDLS), and 40 USTS were used. The CNV plots of all well-differentiated/dedifferentiated liposarcomas showed *MDM2* amplification.

### Differential Methylation Analysis

Differential DNA methylation analysis was performed to identify significantly differentially methylated CpG sites and regions by comparing ∼800,000 CpG sites between responders versus non-responders combining MFS and USTS. Differentially methylated regions were identified using the R-package “DMRcate” (https://github.com/rcavalcante/DMRcate/), with a minimum of 3 differentially methylated CpG islands with a maximal distance between two CpG islands of 500 base pairs. Each region was labeled using the R-package “annotatr” (https://bioconductor.org/packages/release/bioc/html/annotatr.html) in order to identify the proximal promoters of protein-coding genes.

### Copy Number Variation Analysis

Copy number plots were generated from the methylation array data using the R-package “conumee” (http://bioconductor.org/packages/conumee/), as previously described.^16^ The default settings of conumee were used for copy number segmentation^18^ and were as follows: a minimum of probes per bin equal to 50; minimum bin size equal to 100 000 bp. Each CNV plot was manually reviewed. CNVs were called based on a minimum of 5 bins. To identify statistically significant recurrent CNVs, we employed “GISTIC2” (https://broadinstitute.github.io/gistic2/) and the R package “CNsummaryplots” (https://github.com/dstichel/CNsummaryplots). Segmentations produced by conumee served as input for both tools. Fisher's exact test was used to assess significant differences in CNV patterns between responders and non-responders.

## Results

### Patients’ Samples and Clinical Features

Archival tumor specimens from 27 patients diagnosed with MFS and 22 with USTS between 2010 and 2023 at our institute were retrieved. Of the pre-radiotherapy biopsy specimens, 15 yielded insufficient DNA quantities (<100 ng) for methylation analysis. DNA methylation array profiling was performed on the remaining 34 biopsy samples, resulting in interpretable data from 23 tumors (15 MFS and 8 USTS), while 11 samples failed to meet quality control criteria.

Clinicopathologic characteristics of study patients are summarized in Table 1. The 15 patients with MFS included 6 men and 9 women aged 45-85 years old (median, 70 y), with tumors located in the thigh (n = 9), forearm (n = 2), upper arm (n = 2), and knee (n = 2). Tumor sizes ranged from 4.3 to 15.0 cm (median, 7.5 cm). The 8 patients with USTS included 7 men and 1 woman aged 55-88 years old (median, 70.5 y), with tumors located in the thigh (n = 4), shoulder (n = 1), forearm (n = 1), knee (n = 1), and buttock (n = 1). Tumor sizes ranged from 1.7 to 9.0 cm (median, 4.5 cm).

**Table 1.** Clinicopathologic Characteristics of Study Patients.

Patient No.	Sarcoma Subtype	Sex	Tumor Grade	Tumor Site	Tumor Size (cm)	Neoadjuvant Radiotherapy	EORTC-STBSG Response Score^15^
1	MFS	M	2	Thigh	6.4	14 × 3 Gy	E
2	MFS	F	2	Forearm	5.4	25 × 2 Gy	B
3	MFS	F	2	Knee	4.3	25 × 2 Gy	E
4	MFS	F	3	Upper arm	14	25 × 2 Gy	D
5	MFS	F	3	Thigh	13	25 × 2 Gy	C
6	MFS	F	2	Thigh	15.0	25 × 2 Gy	E
7	MFS	F	2	Thigh	11.1	25 × 2 Gy	D
8	MFS	M	2	Upper arm	9.6	14 × 3 Gy	D
9	MFS	M	3	Thigh	7.1	25 × 2 Gy	E
10	MFS	F	2	Thigh	10.0	25 × 2 Gy	D
11	MFS	M	2	Thigh	15.0	25 × 2 Gy	E
12	MFS	F	3	Knee	5.1	25 × 2 Gy	C
13	MFS	M	2	Thigh	7.5	14 × 3 Gy	E
14	MFS	M	3	Thigh	4.4	14 × 3 Gy	D
15	MFS	F	2	Forearm	7.3	25 × 2 Gy	C
16	USTS	M	2	Thigh	4.3	14 × 3 Gy	D
17	USTS	M	3	Forearm	1.7	25 × 2 Gy	E
18	USTS	M	2	Thigh	4.5	14 × 3 Gy	C
19	USTS	M	3	Buttock	9.0	25 × 2 Gy	C
20	USTS	M	3	Thigh	4.9	25 × 2 Gy	E
21	USTS	M	2	Thigh	4.5	14 × 3 Gy	C
22	USTS	M	3	Shoulder	7	14 × 3 Gy	C
23	USTS	F	2	Knee	4.4	14 × 3 Gy	B

Abbreviations: EORTC-STBSG, European Organization for Research and Treatment of Cancer-Soft Tissue and Bone Sarcoma Group; F, female; M, male; MFS, myxofibrosarcoma; USTS, undifferentiated soft tissue sarcoma.

**Figure 1.**
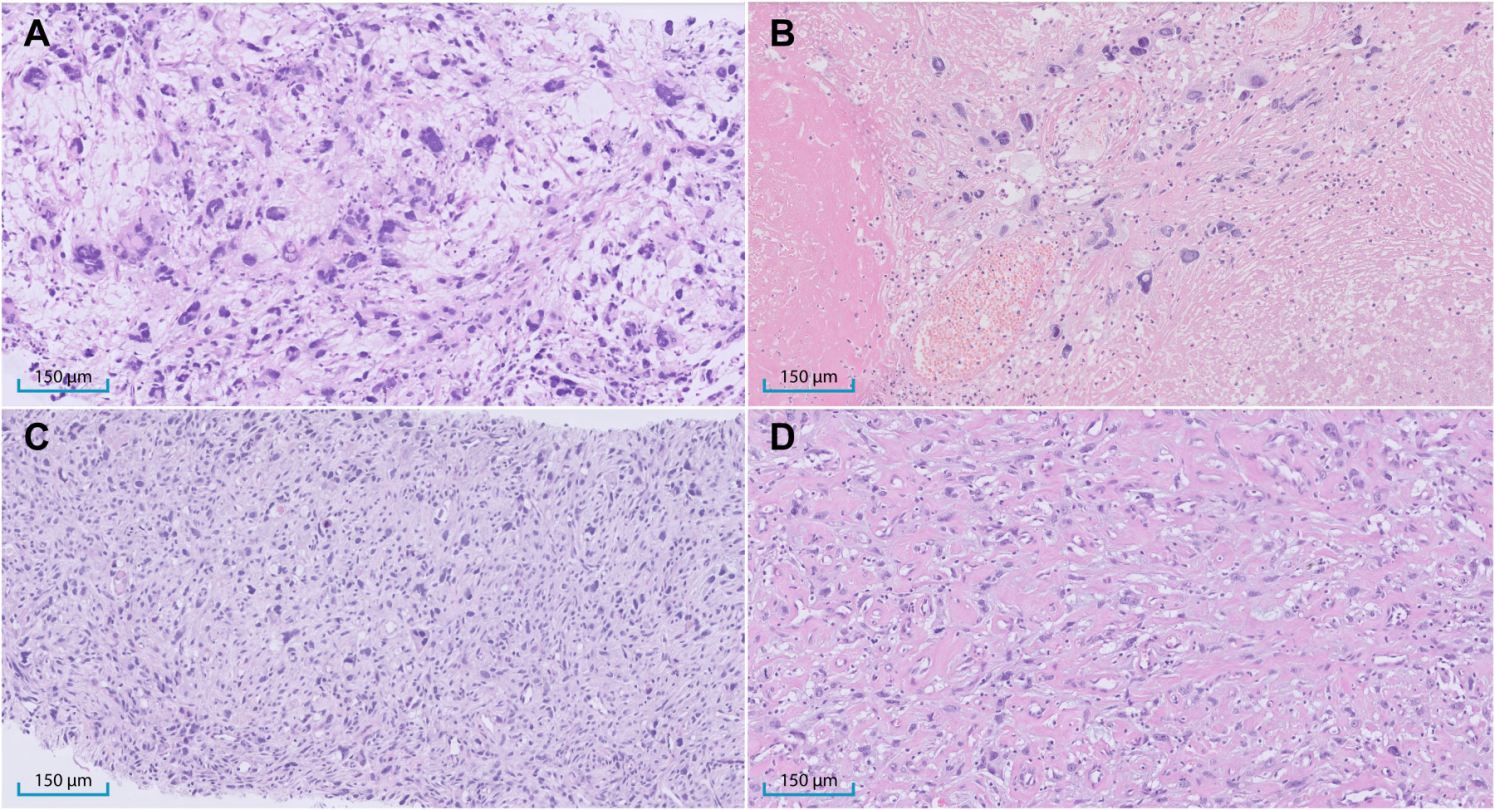
Representative morphological features of myxofibrosarcoma (MFS) and undifferentiated soft tissue sarcoma (USTS) before and after radiotherapy. (A) Pre-radiotherapy biopsy of MFS from patient #5 shows spindled to pleomorphic cells and bizarre multinucleated giant cells embedded in a myxoid stroma. (B) Corresponding post-radiotherapy resection from the same patient shows predominantly fibrotic tissue with areas of necrosis and only sparse residual viable tumor cells (responder; response score C). (C) Pre-radiotherapy biopsy of USTS from patient #16 shows a patternless arrangement of atypical spindled, pleomorphic, and multinucleated cells without myxoid stroma. (D) Corresponding post-radiotherapy resection specimen shows extensive viable tumor areas within a fibrotic background (non-responder; response score D).

All patients received neoadjuvant radiation therapy, with a subset participating in a Dutch randomized phase II clinical trial comparing standard fractionation (25 × 2 Gy) to a hypofractionated regimen (14 × 3 Gy).^19^ Pathologic response scores were as follows: B in 2 patients (9%), C in 7 (30%), D in 6 (26%), and E in 8 (35%), resulting in an overall response rate of 39% (response grades A-C) and 61% classified as non-responders (response grades D-E). Representative histological images of MFS and USTS biopsy and resection specimens are shown in Figure 1.

### Uniform Manifold Approximation and Projection-Based Classification

**Figure 2.**
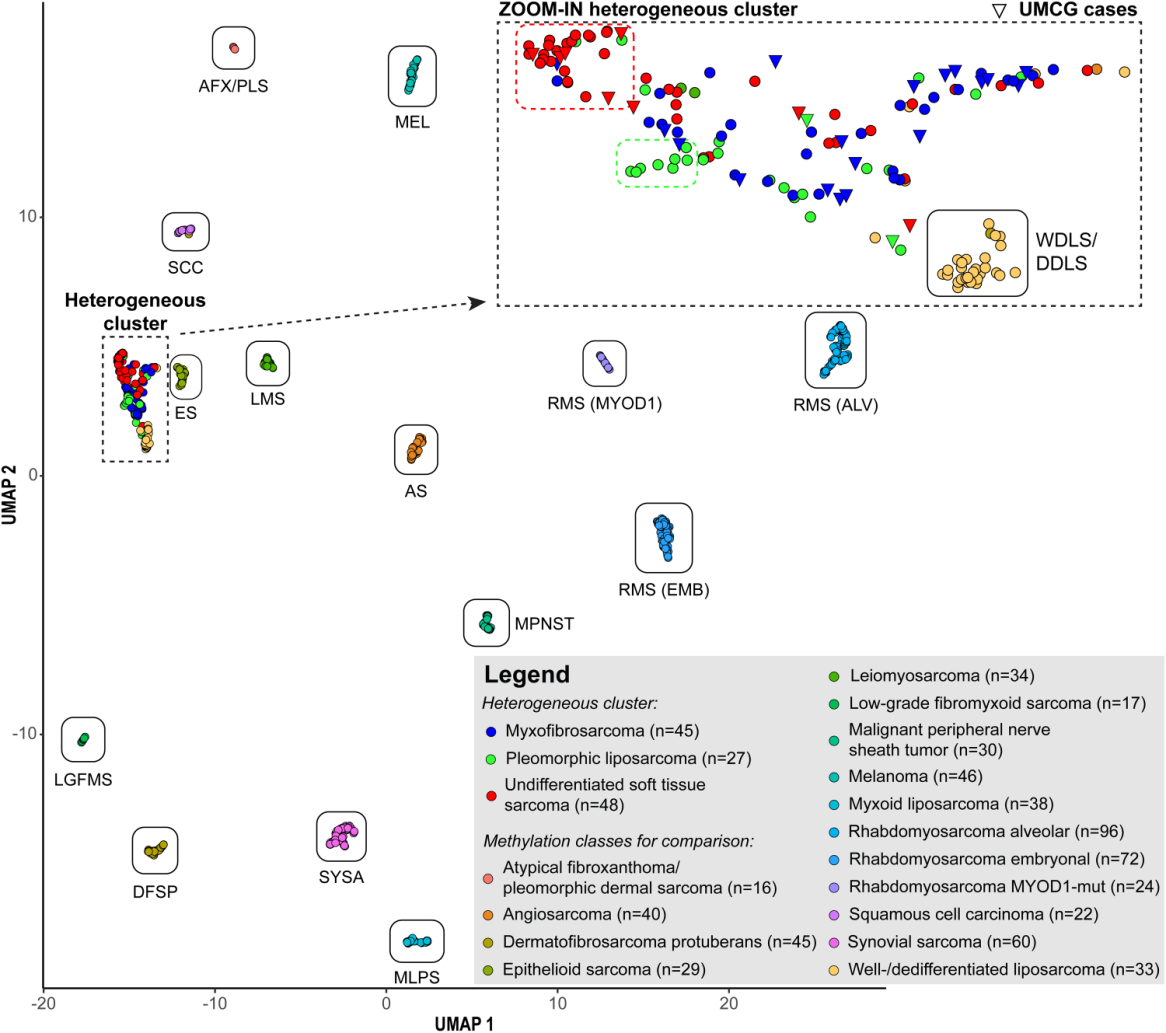
Unsupervised methylation-based clustering of myxofibrosarcoma (MFS) and undifferentiated soft tissue sarcoma (USTS). Cluster analysis was performed using genome-wide DNA methylation data from both the study cohort and publicly available reference datasets.

Unsupervised DNA methylation cluster analysis was performed on our patient cohort combined with publicly available datasets to validate diagnoses. UMAP analysis showed that MFS and USTS together with PLS formed a single heterogeneous cluster. This cluster was distinct yet close to the well-differentiated/dedifferentiated liposarcoma cluster and separated from all other soft tissue tumor types (Figure 2).

### Methylome Analysis to Identify Biomarkers for Radiotherapy Response

**Figure 3.**
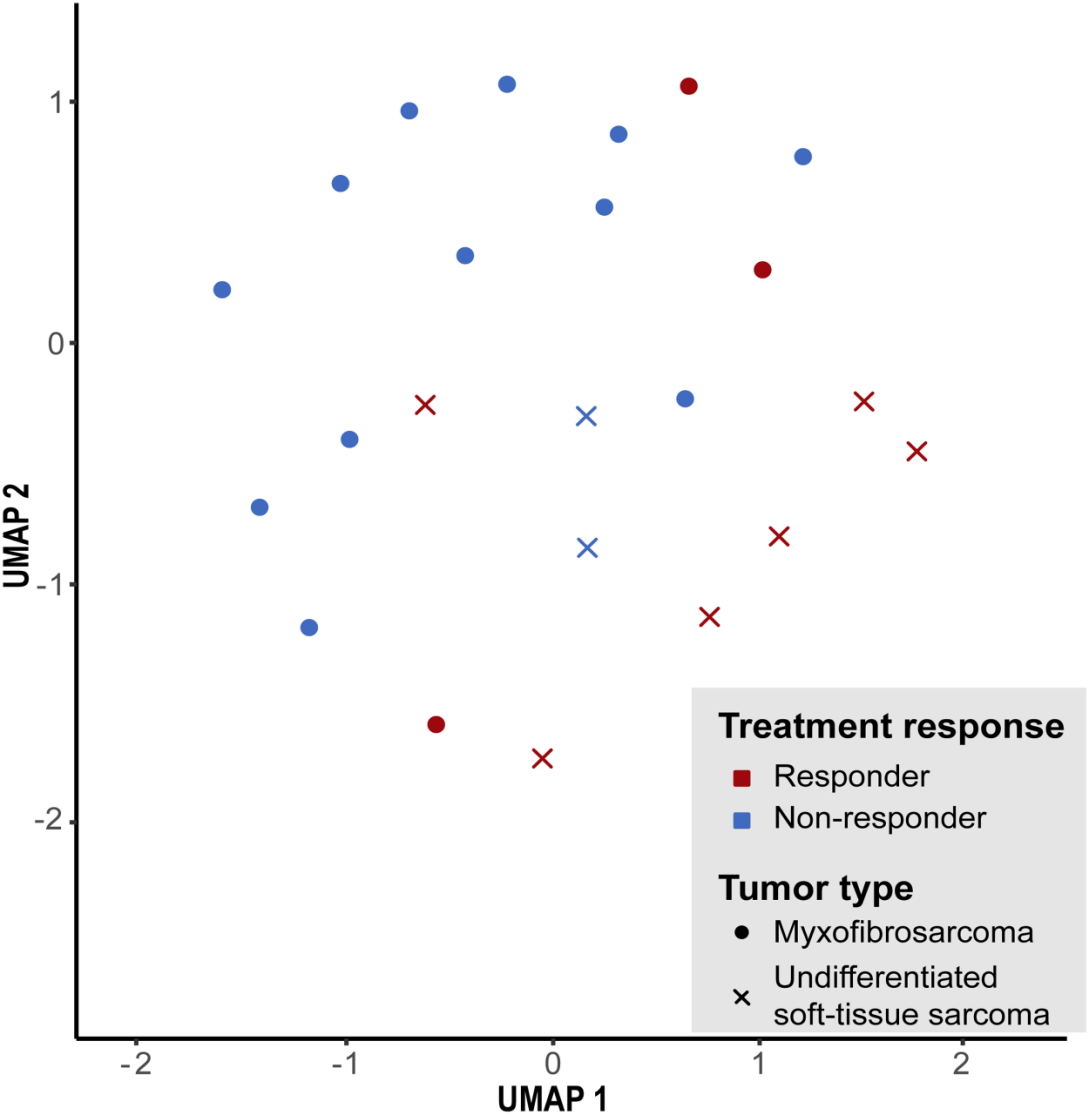
Unsupervised methylation-based clustering responders and non-responders to neoadjuvant radiotherapy. Cluster analysis was performed using genome-wide DNA methylation data derived exclusively from the study cohort. The analysis did not reveal distinct clustering patterns between responders and non-responders.

To investigate meaningful subgroups predictive of radiotherapy response, unsupervised clustering was repeated using only our MFS and USTS methylation data, excluding publicly available datasets due to unknown therapy response. MFS and USTS were combined given their molecular indistinguishability. The analysis revealed that MFS and USTS formed a similarly heterogeneous cluster as shown in Figure 2, with no evidence of distinct subgroups associated with radiotherapy response (Figure 3).

To explore the methylome further, we conducted a differential DNA methylation analysis, comparing ∼800.000 individual CpG sites between responders and non-responders to identify significantly differentially methylated CpG sites and regions. However, this analysis also did not reveal any significant differences in methylation status of individual CpG sites between the two groups (data not shown).

### Copy Number Variation Analysis

**Figure 4.**
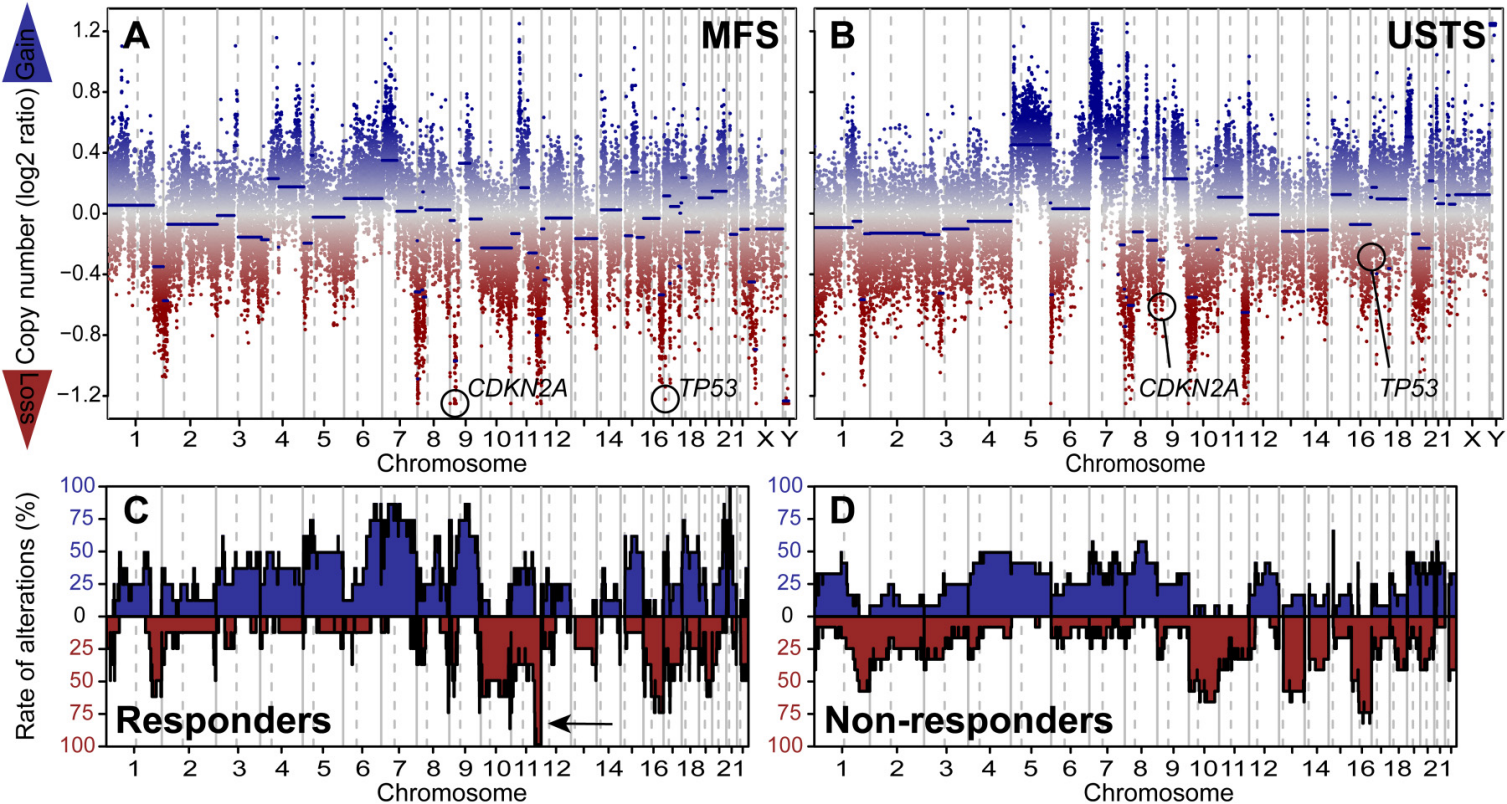
Copy number variation (CNV) analysis. (A) Representative CNV plot of myxofibrosarcoma (MFS) from patient #5 showing multiple gains and losses, including homozygous deletions of *CDKN2A* and *TP53*. (B) Representative CNV plot of undifferentiated soft tissue sarcoma (USTS) from patient #18 showing heterozygous deletions of *CDKN2A* and *TP53*. (C, D) Rates of CNVs in responders and non-responders were largely similar, with the exception of chromosome 11q24.1, which was significantly more frequently lost in responders (indicated by arrow).

Twenty MFS and USTS yielded interpretable CNV plots, all demonstrating complex genomes characterized by multiple gains and losses (Figure 4A and B). The amount of chromosomal instability and CNV distribution did not differ significantly between MFS and USTS. Recurrent copy number gains were observed in chromosomes or chromosome arms 1p, 4, 5, 6q, 7, 9, 12, 19, and 20, while frequent losses were identified in 1q, 8p, 9p, 10, 11q, 13, 16q, 17p, 18q, and 22q. Notably, recurrent losses involved key tumor suppressor genes, including *CDKN2A* (n = 10), *RB1* (n = 5), and *TP53* (n = 9).

The analysis was repeated to evaluate CNV patterns between responders (n = 9) and non-responders (n = 11), revealing no significant difference in the amount of chromosomal instability. The CNV distributions were largely overlapping (Figure 4C and D). However, a statistically significant difference was identified at chromosome 11q24.1, which was deleted in all responders but only in a subset of non-responders (33%; P = 0.0039). This difference was independent of tumor subtype.

## Discussion

In this study, we explored DNA methylation and CNV patterns in MFS and USTS, obtained from pre-radiotherapy biopsy specimens, to identify potential predictive biomarkers for response to neoadjuvant radiotherapy. Our results showed that MFS and USTS share similar DNA methylation and CNV profiles. However, we found no evidence supporting the utility of DNA methylation profiling as a predictive tool for radiotherapy response. CNV patterns were also largely similar between responders and non-responders, with the exception of chromosome 11q24.1 which was significantly more frequently lost in responders than in non-responders.

In recent years, DNA methylation profiling has emerged as a valuable diagnostic tool for confirming and refining histomorphology-based classifications.^20^ The sensitivity and specificity of DNA methylation profiles vary across tumor classes, with fusion-driven neoplasms typically forming well-defined clusters, whereas tumors characterized by genomic complexity and high intratumoral heterogeneity tend to display more ambiguous patterns.^17^ In our analysis, unsupervised clustering showed that MFS and USTS formed a single heterogeneous cluster, indicative of overlapping DNA methylation signatures. This finding aligns with the Heidelberg sarcoma classifier, which groups both MFS and USTS within the “undifferentiated sarcoma” (USARC) methylation class, alongside pleomorphic liposarcoma.^11^ Notably, a closer examination of our clustering results revealed a tendency for USTS and PLS to form distinct subclusters, although the separation was not definitive. Whether this observation reflects true underlying biological differences remains unclear and future studies with larger cohorts may provide more clarity.

The success of recently developed DNA methylation-based classifiers underscores the biological relevance of DNA methylation in cancer, as it reflects key features such as cellular differentiation, gene expression profiles, clinical behavior, and therapeutic response.^21^ A well-established example of methylation serving as a predictive biomarker is the methylation status of the O-6-methylguanine-DNA methyltransferase promoter, which predicts response to alkylating agents like temozolomide in glioblastoma.^22^ In the present study, however, we did not identify significant differences in the methylation status of individually CpG sites between responders and non-responders, suggesting that DNA methylation alone is unlikely to serve as a reliable biomarker for predicting radiotherapy response in MFS and USTS.

In contrast, CNV analysis identified a potentially significant genomic alteration: loss of chromosomal region 11q24.1 in all treatment responders, compared to only 33% of non-responders. This region contains 270 genes (Supplemental Table 1), including Ataxia telangiectasia mutated (*ATM*), which is well known for its role in repairing double-strand DNA breaks within the DNA damage response pathway.^23,24^ *ATM* is activated via autophosphorylation at Ser1981 in response to double-strand DNA breaks, initiating a phosphorylation cascade that leads to DNA repair and cell cycle checkpoint control.^25^ A future study investigating *ATM* expression in MFS and USTS—for example, through immunohistochemical analysis—would therefore be of great interest to determine whether responders exhibit lower *ATM* expression compared to non-responders.

Whether this recurrent loss of 11q24.1 represents a true predictive biomarker or merely a coincidental finding due the extensive chromosomal instability characteristic of MFS and USTS remains uncertain. Validation in larger cohorts is necessary to determine its predictive value. Future studies aiming to investigate CNVs more robustly should consider employing high-resolution methods, such as shallow whole-genome sequencing, which offers greater sensitivity than CNV plots generated from DNA methylation data. Moreover, CNV plots derived from methylation arrays do not provide information on tumor ploidy status, which is frequently altered in MFS and USTS, with triploid and tetraploid genomes commonly observed.^26^

A limitation of this study is the small sample size, which is partly attributable to a high dropout rate. Of the 49 patients included, 15 biopsy specimens yielded insufficient DNA quantities (<100 ng) for methylation analysis. DNA methylation profiling generated interpretable results in 23 of the remaining 34 samples. Notably, the Illumina FFPE DNA Restoration Kit was not utilized in the initial 23 samples, which contributed to the high dropout rate. Implementation of the restoration protocol in the subsequent 11 samples substantially improved assay success. Reanalysis of the first 23 samples was not performed due to financial constraints and limited availability of residual tissue material. Notably, archival specimens older than 3 years were more likely to fail, whereas more recently acquired samples showed significantly higher success rates (data not shown), supporting the feasibility of DNA methylation profiling in clinical practice. Additionally, while baseline epigenetic differences may influence radiosensitivity, radiotherapy itself may also induce specific DNA methylation changes that affect tumor cell survival and radiosensitivity.^^27^^ In this study, we did not analyze such potentially treatment-induced changes.

In conclusion, our results support the concept that MFS and USTS represent a spectrum of the same disease indicating that the amount of myxoid stroma is merely a morphological variation without molecular and/or biological significance. Although DNA methylation profiling does not appear to be predictive for response to neoadjuvant radiotherapy, the loss of chromosome 11q24.1 in responders warrants further investigation as a potential biomarker for radiosensitivity.

## Supplemental Material

sj-docx-1-ijs-10.1177_10668969251412902 - Supplemental material for Radiotherapy Response Prediction in Myxofibrosarcomas and Undifferentiated Soft Tissue Sarcomas Using DNA Methylation and Copy Number Profiling Supplemental material, sj-docx-1-ijs-10.1177_10668969251412902 for Radiotherapy Response Prediction in Myxofibrosarcomas and Undifferentiated Soft Tissue Sarcomas Using DNA Methylation and Copy Number Profiling by Tony G. Kleijn, Baptiste Ameline, Wierd Kooistra, Léon C. van Kempen, Gilles F.H. Diercks and Robert J. van Ginkel, Lukas B. Been, Barbara L. van Leeuwen, Anna K.L. Reyners, Thomas C. Kwee, Paul C. Jutte, Joris J.W. Ploegmakers, Henk Bijl, Bart Vanhauten, J. Fred Ubbels, Ed Schuuring, Albert J.H. Suurmeijer, Jacco J. de Haan, Daniel Baumhoer, Arjen H.G. Cleven in International Journal of Surgical Pathology

## References

[1] WHO Classification of Tumours Editorial Board. Soft Tissue and Bone Tumours. International Agency for Research on Cancer; 2020. (WHO Classification of Tumours Series, 5th Ed.; Vol. 3). Https://Publications.Iarc.Fr/588 .

[2] Yoshimoto M Yamada Y Ishihara S , et al. Comparative study of myxofibrosarcoma with undifferentiated pleomorphic sarcoma. Am J Surg Pathol. 2020;44(1):87-97. 31651522 10.1097/PAS.0000000000001389

[3] Lee AY Agaram NP Qin LX , et al. Optimal percent myxoid component to predict outcome in high-grade myxofibrosarcoma and undifferentiated pleomorphic sarcoma. Ann Surg Oncol. 2016;23(3):818-825. 26759307 10.1245/s10434-015-5063-5 PMC4964786

[4] Cancer Genome Atlas Research Network. Comprehensive and integrated genomic characterization of adult soft tissue sarcomas. Cell*.* 2017;171(4):950-965.e28. 29100075 10.1016/j.cell.2017.10.014 PMC5693358

[5] Sun H Liu J Hu F , et al. Current research and management of undifferentiated pleomorphic sarcoma/myofibrosarcoma. Front Genet. 2023;14:1109491. 36873946 10.3389/fgene.2023.1109491 PMC9978151

[6] Mitra S Farswan A Piccinelli P , et al. Transcriptomic profiles of myxofibrosarcoma and undifferentiated pleomorphic sarcoma correlate with clinical and genomic features. J Pathol. 2024;264(3):293-304. 39258383 10.1002/path.6347

[7] O'Sullivan B Davis AM Turcotte R , et al. Preoperative versus postoperative radiotherapy in soft-tissue sarcoma of the limbs: a randomised trial. Lancet. 2002;359(9325):2235-2241. 12103287 10.1016/S0140-6736(02)09292-9

[8] Sampath S Schultheiss TE Hitchcock YJ , et al. Preoperative versus postoperative radiotherapy in soft-tissue sarcoma: multi-institutional analysis of 821 patients. Int J Radiat Oncol Biol Phys*.* 2011;81(2):498-505. 20888702 10.1016/j.ijrobp.2010.06.034

[9] El-Bared N Wong P Wang D . Soft tissue sarcoma and radiation therapy advances, impact on toxicity. Curr Treat Options Oncol. 2015;16(5):19. 25859829 10.1007/s11864-015-0335-7

[10] Price JM Prabhakaran A West CML . Predicting tumour radiosensitivity to deliver precision radiotherapy. Nat Rev Clin Oncol. 2023;20(2):83-98. 36477705 10.1038/s41571-022-00709-y

[11] Koelsche C Schrimpf D Stichel D , et al. Sarcoma classification by DNA methylation profiling. Nat Commun. 2021;12(1):498. 33479225 10.1038/s41467-020-20603-4 PMC7819999

[12] Cheng L Pandya PH Liu E , et al. Integration of genomic copy number variations and chemotherapy-response biomarkers in pediatric sarcoma. BMC Med Genet. 2019;12(Suppl 1):23. 10.1186/s12920-018-0456-5 PMC6357363 30704460

[13] Zhu X Wang Y Tan L Fu X . The pivotal role of DNA methylation in the radio-sensitivity of tumor radiotherapy. Cancer Med. 2018;7(8):3812-3819. 29952116 10.1002/cam4.1614 PMC6089158

[14] Baumann M Krause M Dikomey E , et al. EGFR-targeted anti-cancer drugs in radiotherapy: preclinical evaluation of mechanisms. Radiother Oncol. 2007;83(3):238-248. 17502118 10.1016/j.radonc.2007.04.006

[15] Wardelmann E Haas RL Bovée JV , et al. Evaluation of response after neoadjuvant treatment in soft tissue sarcomas; the European organization for research and treatment of cancer-soft tissue and bone sarcoma group (EORTC-STBSG) recommendations for pathological examination and reporting. Eur J Cancer. 2016;53:84-95. 26700077 10.1016/j.ejca.2015.09.021

[16] Kleijn TG Ameline B Schreuder WH , et al. Odontogenic myxomas harbor recurrent copy number alterations and a distinct methylation signature. Am J Surg Pathol. 2024;48(10):1224-1232. 39289817 10.1097/PAS.0000000000002293

[17] Lyskjaer I De Noon S Tirabosco R , et al. DNA methylation-based profiling of bone and soft tissue tumours: a validation study of the ‘DKFZ Sarcoma Classifier’. J Pathol Clin Res. 2021;7(4):350-360. 33949149 10.1002/cjp2.215 PMC8185366

[18] Daenekas B Pérez E Boniolo F , et al. Conumee 2.0: enhanced copy-number variation analysis from DNA methylation arrays for humans and mice. Bioinformatics*.* 2024;40(2):btae029. 10.1093/bioinformatics/btae029 PMC10868300 38244574

[19] Short Course of Preoperative Radiotherapy in Head and Neck-, Trunk- and Extremity Soft Tissue Sarcomas (SCOPES) . ClinicalTrials.gov identifier: NCT04425967. Updated December 12, 2024. Accessed May 5, 2025. https://clinicaltrials.gov/study/NCT04425967. 10.1136/bmjopen-2026-117842 PMC13629992 42778256

[20] Koelsche C von Deimling A . Methylation classifiers: brain tumors, sarcomas, and what's next. Genes, Chromosomes and Cancer*.* 2022;61(6):346-355. 35388566 10.1002/gcc.23041

[21] Papanicolau-Sengos A Aldape K . DNA methylation profiling: an emerging paradigm for cancer diagnosis. Annu Rev Pathol: Mech Dis. 2022;17:295-321. 10.1146/annurev-pathol-042220-022304 34736341

[22] Gilbert MR Wang M Aldape KD , et al. Dose-dense temozolomide for newly diagnosed glioblastoma: a randomized phase III clinical trial. J Clin Oncol. 2013;31(32):4085-4091. 24101040 10.1200/JCO.2013.49.6968 PMC3816958

[23] Pitter KL Casey DL Lu YC , et al. PathogenicATM mutations in cancer and a genetic basis for radiotherapeutic efficacy. J Natl Cancer Inst*.* 2021;113(3):266-273. 32726432 10.1093/jnci/djaa095 PMC7936050

[24] Kim KH Kim HS Kim SS , et al. Increased radiosensitivity of solid tumors harboring ATM and BRCA1/2 mutations. Cancer Res Treat. 2022;54(1):54-64. 34082492 10.4143/crt.2020.1247 PMC8756123

[25] Jin MH Oh DY . ATM in DNA repair in cancer. Pharmacol Ther. 2019;203:107391. 31299316 10.1016/j.pharmthera.2019.07.002

[26] Nishio J Nakayama S . Biology and management of high-grade myxofibrosarcoma: state of the art and future perspectives. Diagnostics (Basel)*.* 2023;13(19):3022. 37835765 10.3390/diagnostics13193022 PMC10572210

[27] Wang Y Han Y Jin Y He Q Wang Z . The advances in epigenetics for cancer radiotherapy. Int J Mol Sci. 2022;23:5654. 35628460 10.3390/ijms23105654 PMC9145982

