# Supplementary material for: Radiotherapy Response Prediction in Myxofibrosarcomas and Undifferentiated Soft Tissue Sarcomas Using DNA Methylation and Copy Number Profiling
Source: Int J Surg Pathol. 2026 Jan 29;34(4):920–8. doi: 10.1177/10668969251412902 (PMC13168605; doi:10.1177/10668969251412902)
Supplement: sj-docx-1-ijs-10.1177_10668969251412902 - Supplemental material for Radiotherapy Response Prediction in Myxofibrosarcomas and Undifferentiated Soft Tissue Sarcomas Using DNA Methylation and Copy Number Profiling [file sj-docx-1-ijs-10.1177_10668969251412902.docx]

**Supplemental Table 1.** Genes on chromosome 11q24.1.

| *ACAT1* | *ACRV1* | *APLP2* | *APOA1* | *APOA4* | *APOC3* |
| --- | --- | --- | --- | --- | --- |
| *ARCN1* | *ATM* | *FXYD2* | *CXCR5* | *CBL* | *CD3D* |
| *CD3E* | *CD3G* | *CHEK1* | *CRYAB* | *DDX6* | *DDX10* |
| *DLAT* | *DPAGT1* | *DRD2* | *ETS1* | *FDX1* | *FLI1* |
| *SLC37A4* | *GRIK4* | *GUCY1A2* | *H2AFX* | *HMBS* | *HSPA8* |
| *HSPB2* | *HTR3A* | *IL10RA* | *IL18* | *STT3A* | *KCNJ1* |
| *KCNJ5* | *VWA5A* | *MCAM* | *KMT2A* | *NCAM1* | *NFRKB* |
| *NNMT* | *NPAT* | *NRGN* | *OPCML* | *PAFAH1B2* | *POU2AF1* |
| *PPP2R1B* | *PTS* | *NECTIN1* | *RDX* | *RPS25* | *SC5D* |
| *SCN2B* | *SCN4B* | *SDHD* | *ST3GAL4* | *SLN* | *SORL1* |
| *SRPRA* | *ST14* | *TAGLN* | *TECTA* | *THY1* | *UPK2* |
| *ZBTB16* | *ZNF202* | *CUL5* | *BARX2* | *ZPR1* | *USP2* |
| *PCSK7* | *HTR3B* | *ZW10* | *UBE4A* | *EI24* | *FEZ1* |
| *ARHGAP32* | *C2CD2L* | *RBM7* | *MPZL2* | *HYOU1* | *ATP5L* |
| *ADAMTS8* | *TREH* | *CEP164* | *IGSF9B* | *EXPH5* | *PHLDB1* |
| *SIK2* | *NCAPD3* | *ARHGEF12* | *SIK3* | *VSIG2* | *BACE1* |
| *TRIM29* | *CADM1* | *POU2F3* | *HINFP* | *REXO2* | *OR8G2* |
| *OR8B8* | *OR8G1* | *TIMM8B* | *OR8B2* | *ACAD8* | *B3GAT1* |
| *DCPS* | *ZBTB44* | *THYN1* | *DDX25* | *NTM* | *CDON* |
| *SIDT2* | *TRAPPC4* | *SPA17* | *FXYD6* | *SIAE* | *C11orf71* |
| *ROBO4* | *SLC35F2* | *RAB39A* | *BTG4* | *NXPE4* | *TTC12* |
| *C11orf57* | *ELMOD1* | *FOXRED1* | *SCN3B* | *VPS11* | *TEX12* |
| *CRTAM* | *TMPRSS4* | *IFT46* | *PRDM10* | *DSCAML1* | *GRAMD1B* |
| *ARHGAP20* | *USP28* | *PKNOX2* | *TP53AIP1* | *ABCG4* | *ROBO3* |
| *C11orf1* | *RNF26* | *FAM118B* | *NLRX1* | *MSANTD2* | *ALG9* |
| *CLMP* | *PDZD3* | *C11orf63* | *CCDC15* | *TMPRSS5* | *PUS3* |
| *JAM3* | *BCO2* | *TMPRSS13* | *KIRREL3* | *BUD13* | *TMEM25* |
| *RPUSD4* | *TBRG1* | *UBASH3B* | *SNORD14C* | *SNORD14D* | *SNORD14E* |
| *DIXDC1* | *ZC3H12C* | *GLB1L2* | *ESAM* | *ALKBH8* | *FDXACB1* |
| *C11orf52* | *VPS26B* | *GLB1L3* | *TIRAP* | *C1QTNF5* | *PANX3* |
| *APOA5* | *TMEM45B* | *COLCA2* | *PIH1D2* | *NXPE1* | *NXPE2* |
| *JAML* | *CWF19L2* | *KDELC2* | *LAYN* | *TTC36* | *PATE1* |
| *C11orf65* | *ADAMTS15* | *MPZL3* | *C11orf45* | *HYLS1* | *TMEM218* |
| *SLC37A2* | *OR8B12* | *OR8G5* | *OR10G8* | *OR10G9* | *OR10S1* |
| *OR6T1* | *OR4D5* | *TBCEL* | *TMEM136* | *SPATA19* | *HEPACAM* |
| *OAF* | *ANKK1* | *RNF214* | *LOC283140* | *LINC00900* | *BCL9L* |
| *FOXR1* | *CCDC153* | *OR8D1* | *OR8D2* | *OR8B4* | *KIRREL3-AS3* |
| *C11orf44* | *MIR4697HG* | *LOC283177* | *CCDC84* | *TMEM225* | *OR8D4* |
| *C11orf53* | *LOC341056* | *PLET1* | *LOC387810* | *BSX* | *OR6X1* |
| *OR6M1* | *OR10G4* | *OR10G7* | *OR8B3* | *OR8A1* | *C11orf87* |
| *COLCA1* | *C11orf88* | *MIR100HG* | *PATE2* | *PATE4* | *ST3GAL4-AS1* |
| *SNX19* | *LOC403312* | *MIRLET7A2* | *BLID* | *LINC00167* | *HEPN1* |
| *LOC643923* | *CLDN25* | *RPL23AP64* | *PATE3* | *NCAM1-AS1* | *BACE1-AS* |
| *USP2-AS1* | *SENCR* | *TMPRSS4-AS1* | *HSPB2-C11orf52* | *FXYD6-FXYD2* | *KIRREL3-AS2* |
| *NTM-IT* | *LINC01395* | *NTM-AS1* | *PKNOX2-AS1* | *APOA1-AS* | *STT3A-AS1* |
